# Exploring the Role of Licorice and Its Derivatives in Cell Signaling Pathway NF-*κ*B and MAPK

**DOI:** 10.1155/2024/9988167

**Published:** 2024-10-23

**Authors:** Ieaman Fatima, Amna Sahar, Amna Tariq, Tabana Naz, Muhammad Usman

**Affiliations:** ^1^National Institute of Food Science and Technology, University of Agriculture, Faisalabad 38000, Pakistan; ^2^Department of Food Engineering, University of Agriculture, Faisalabad 38000, Pakistan; ^3^School of Food and Agriculture Science, University of Management and Technology, Lahore, Pakistan

**Keywords:** bioactive compound, cell pathway, inflammation, licorice, MAPK, NF-*κ*B

## Abstract

Licorice is a therapeutic herb in traditional Chinese herbal medicine. Licorice is considered as an anti-inflammatory agent due to its suppression and inhibition of inflammatory pathways. Licorice has many bioactive compounds such as glycyrrhetinic acid, glycyrrhizin, liquiritigenin, and isoliquirtigenin which are principally accountable for its therapeutic benefits. These bioactive components reduce inflammation by preventing the activation of important inflammatory pathways including mitogen-activated protein kinases (MAPKs) and nuclear factor-kappa B (NF-*κ*B). As a result of this tumor necrosis factor-alpha (TNF-*α*), interleukin-1 beta (IL-1*β*) and interleukin-6 (IL-6) are among the proinflammatory cytokines whose production is inhibited. Components present in licorice inhibit the activation by suppressing the I*κ*B*α* phosphorylation and degradation. Moreover, licorice compounds also attenuate the MAPK signaling cascades by inhibiting the MAPK kinase phosphorylation and downstream MAPKs such as extracellular signal–regulated kinase (ERK), p38 MAPK, and c-Jun N-terminal kinase (JNK). The present review focuses on the current understanding of licorice effect on the NF-*κ*B and MAPK inflammatory cell signaling pathways at molecular level. Furthermore, emerging evidence suggested that licorice-derived bioactive compounds may attenuate the molecular mechanism which is associated with inflammation, providing the additional insights into the therapeutic potential. Further studies explained the precise molecular mechanism at the cellular level underlying the licorice anti-inflammatory effect and potential application in managing inflammatory disorders. In conclusion, licorice has a complex mode of action and is a valuable natural anti-inflammatory. Its natural origin and effectiveness in clinical applications make it an intriguing topic for additional study. As licorice becomes more widely used in medicine, future research should focus on refining its formulations to optimize therapeutic advantages.

## 1. Introduction

Emerging domains in clinical medicine are emphasizing the therapeutic potential of natural chemicals and medicinal plants. Licorice, a well-known ancient herb, is often used in traditional Chinese medicine. According to Chinese Pharmacopoeia, the three original Leguminosae plants that are suitable for the manufacturing of licorice are *Glycyrrhiza glabra* L., *Glycyrrhiza inflata Bat*., and Glycyrrhiza uralensis Fisch. Medications prescribed for clinical use frequently require making cut into the rhizome and root of the licorice plant [1]. This therapeutic plant belongs to the Fabaceae family which is also known as Leguminosae, and its derivatives are commonly used as medicinal food [1]. Licorice root has a variety of derivatives, including saponins such as glycyrrhizin, which is 60 times sweeter than sugarcane. It also contains a variety of flavonoids such as liquiritigenin, glucoliquiritin apioside, prenyllicoflavone A, licopyranocoumarin, glisoflavone, liquiritin, 1-metho-xyphaseolin, shinpterocarpin, coumarin-GU-12, rhamnoliquirilin, shinflavanone, and licoarylcoumarin [2]. Furthermore, it has been established that there are four isoprenoid-substituted phenolic components such as terpineol, kanzonol R (a prenylated isoflavan derivative), licoriphenone, 1-methoxyficifolinol, *α*-terpineol, geraniol, as well as linalool oxide A and B. Additional components that have been reported are terpinen-4-ol, kanzonol R, licoriphenone, isoangustone A, 1-methoxyficifolinol, and semilicoisoflavone B. In order to produce maltol, 1-methyl-2-formylpyrrole, 2, 3-butanediol, trimethylpyrazine, furfuraldehyde, methyl ethyl ketone, and furfuryl formate, these compounds were isolated from essential oil. In *G. glabra*, the saponin glycyrrhizin and the aglycone glycyrrhetinic acid are two important compounds, responsible for their therapeutic effects [2, 3]. Clinically, it is utilized for the treatment of various conditions affecting the respiratory, digestive, and immunological systems [4–8] and does not have any substantial negative impacts reported so far. In addition, chemicals derived from licorice have anticarcinogenic, anti-inflammatory, and antiviral properties [9–11]. Thus, these pharmacological and therapeutic properties help against inflammatory disease treatment [12–14]. Furthermore, licorice roots exhibit various pharmacological effects and have many medical applications, including antiulcer, anti-inflammatory, antispasmodic, and antidepressive properties. Other potential therapeutic effects are antiviral, antioxidant, hypoglycemic, and hepatoprotective [15, 16].

There are a number of chronic diseases in which inflammation is a common pathophysiological mechanism [17]. These diseases include cancer, inflammatory bowel disease, heart disease, and diabetes. The mechanisms that are responsible for the inflammatory response inside the body are determined by the precise nature of the initial stimulus along with its location. Following the recognition of harmful stimuli by cell surface pattern receptors, three successive steps take place: (1) the generation of inflammatory mediators; (2) the recruitment of inflammatory cells; and (3) the stimulation of inflammatory pathways [18].

Numerous chronic diseases exhibit regulatory mechanisms and inflammatory mediators that play crucial roles in the responsible inflammatory pathways. Intracellular signaling pathways are activated by the inflammatory stimuli which lead to the subsequent stimulation of inflammatory mediator production. The interleukin-1 receptor (TNFR) is responsible for inducing inflammation through the interaction with various interleukins and cytokines, such as interleukin-1 (IL-1)), interleukin-1 receptor (IL-1R), and interleukin-6 (IL-6) receptor (IL-6R). These interleukins and cytokines include microbial products, tumor necrosis factor-*γ*, IL-6, and interleukin-1*β* (IL-1*β*) [19]. Receptor activation is responsible for the activation of mitogen-activated protein kinase (MAPK), activator of transcription (STAT), and Janus kinase (JAK) signal transducer, which are important intracellular signaling pathways [20, 21].

It was shown in a study that 5-(1,1-dimethylallyl)− 3,4,4′ -trihydroxy-2-methoxychalcone, licochalcone A (LicoA) and B, echinatin, and glycycoumarin are the derivatives of licorice which had the capacity to suppress the formation of NO, IL-6, and prostaglandins (PGE2) in the quest for a bioactive anti-inflammatory moiety [22]. Furthermore, Li et al. [23] reported the observation of mRNA and protein expressions related to the inhibition of inducible nitric oxide synthase (iNOS), cyclooxygenase 2 (COX-2), cytokines, tumor necrosis factor-alpha (TNF-*α*), IL-1*β*, and IL-6 production by the licorice derivatives. Similar findings were made regarding the inhibition of lipopolysaccharide (LPS)-induced proinflammatory mediator increase, including iNOS, COX-2, TNF-*α*, IL-1*β*, and IL-6 in BV2 cell line, by bioactive substances such as liquiritin, glycyrrhizic acid, and liquiritigenin. However, it was shown that in the liver of mice given t-BHP, licorice extract inhibited the production of proinflammatory cytokines such IL-1*β*, TNF-*α*, and IL-6 [24]. Later, a similar study on licorice extract and its derivatives demonstrated anti-inflammatory effect by suppressing the nuclear factor kappa B (NF-*κ*B)–dependent genes including TNF, PGE2, matrix metalloproteinases (MMPs), and the cascade of free radicals [25].

It was discovered that the root extract considerably inhibited the proinflammatory cascade, making it more cytoprotective than the leaf extract [26]. A recent research further supported the notion that therapy with *G. glabra* decreased the levels of IL-5, GTP, IL-13, GOT (on Day 51), mRNA expression of eotaxin, CCL11, CCL24, COX-2, mucus production, eosinophil infiltration, and goblet cell hyperplasia [27]. In conclusion, glycyrrhetinic acid (GA) and glycyrrhizin suppress the production of reactive oxygen species (ROS) and effectively prevent tissue inflammation by blocking the production of IL-3, IL-5, IL-6, IL-10, IL-12, IL-13, IL-1*β*, TNF-*α* expression, COX-2, and eotaxin [12].

### 1.1. NF-*κ*B Signaling Pathway

Through the regulation of the expression of hundreds of genes, NF-*κ*B proteins can regulate different important physiological processes, such as inflammation, cell death, and proliferation [20]. NF-*κ*B proteins can be considered regulators of cellular homeostasis due to the activity of NF-*κ*B in response to a wide variety of stimuli [28]. The ability of NF-*κ*B to modulate the immune response at different stages is crucial for the immune system and inflammation to work together for appropriate and integrated functioning of the human body. Specific cell types including macrophages and dendritic cells facilitated the identification of pathogens during the initial line of defense, through the examination of pathogen-associated molecular patterns (PAMPs), referred to as the innate immune response [29]. These receptors include Toll-like receptor 4 (TLR4) and other members of the Toll-like receptor (TLR) family. The activation of the NF-*κ*B protein occurs when the bacterial LPS is identified by the TLR4 receptor [30].

NF-*κ*B proteins can modify inflammation, which is one of their key activities in the presence of inflammation, which indicates that they target the complex defense mechanisms of the body under inflammation conditions [31]. This is performed by positively and negatively regulating numerous essential genes, including those encoding chemokines and proinflammatory cytokines. IL-1*β* and TNF-*α* are significant cytokines that can activate NF-*κ*B [32]. Furthermore, NF-*κ*B is involved in the resolution of inflammation, a process that disrupts NF-*κ*B functionality [33, 34].

#### 1.1.1. NF-*κ*B Mechanism in Inflammation

To regulate the immune and inflammatory responses, some crucial transcription factors belong to NF-*κ*B family [35]. There are five structurally related proteins such as NF-*κ*B1 (p50), NF-*κ*B2 (p52), RelA (p65), RelB, and c-Rel which are involved in the NF-*κ*B pathway. For the regulation of targeted gene transcription, these proteins produced different homo and heterodimers which bind with unique DNA compounds (*κ*B enhancers) [36]. There are two major pathways (canonical and noncanonical pathways) involved; despite their different mechanism, each pathway plays a significant role in the immune system. In the inactive state, inhibitor proteins (I*κ*B*α*) sequestered NF-*κ*B dimers in the cytoplasm. Translocation of NF-*κ*B to nucleus as well as binding to DNA is prevented by this inhibition. There are various stimuli such as T-cell receptor (TCR), TNFR superfamily members, cytokines (TNF-*α* and IL-1), and pattern recognition receptors (PRRs) to activate the canonical pathway. I*κ*B kinase (IKK) complex is the core of the canonical pathway which is composed of two catalytic subunits (IKK*α* and IKK*β*) along with a regulatory subunit IKK*γ* [37]. IKK complex is activated by various stimuli; IKK phosphorylates I*κ*B*α* on certain serine sites during activation. I*κ*B*α* that has been phosphorylated is ubiquitinated, which causes the proteasome to degrade it. NF-*κ*B dimers, mainly p50/RelA and p50/c-Rel, are released from the inhibitory complex by the degradation of I*κ*B*α*, enabling their translocation to the nucleus. Then in the nucleus, NF-*κ*B binds with *κ*B enhancer elements in the DNA and initiates the gene transcriptions involved in cell proliferation, immune response, inflammation, and survival [38], while in the noncanonical pathway, protein p100 is a precursor and I*κ*B-like inhibitor and inactivates the NF-*κ*B complexes in the cytoplasm. Specific stimuli such as TNFR family member's ligands (CD40, BAFFR, LT*β*R, and RANK) activated this pathway. NF-*κ*B-inducing kinase (NIK) is the center of the noncanonical pathway. NIK is activated by the upstream signals, and then this activated NIK further activates the IKK*α*. This IKK*α* then phosphorylates the 100 protein which leads to its partial degradation. It results in the removal of inhibitory C-terminal domain and produces the developed p52 subunit. These p52/RelB dimers transfer to nucleus. In the nucleus, p52/RelB dimers bind with *κ*B enhancer elements and regulate the genes that are responsible for the immune response, bone metabolism, and lymphoid organ development. In the canonical pathway, NF-*κ*B regulates the gene expressions that encode the inflammatory mediators, responsible for both acute and chronic inflammation. The canonical pathway is complimented by the noncanonical pathway, specifically in adaptive immunity. Noncanonical pathway regulates the function of certain immune cells such as dendritic cells and B cells as well as regulates the development of lymphoid organs [37, 38].

### 1.2. MAPK Pathway

Protein kinases that are activated by mitogen phosphorylate are the serine and threonine residues of their substrates (autophosphorylation) and dual serine [39]. This allows them to either activate or deactivate their target. As a consequence of this, MAPKs are responsible for controlling essential physiological functions such as cell proliferation, responses to stress, cell death, and immune response [40–42]. MAPKs have been identified in numerous eukaryotic organisms [39, 43]. Following a prior stimulation, each MAPK is phosphorylated by an upstream MAPK, thereby initiating an MAPK cascade via a sequential phosphorylation module. MAPK modules commence with an MAP3K, which in turn initiates an MAP2K, resulting in the activation of MAPK [39, 43, 44]. MAPK protein phosphatases (MKPs) are capable of dephosphorylating phosphothreonine and phosphotyrosine residues on MAPKs [41, 45], thereby leaving inactive the phosphorylation processes of these proteins. Mammalian cells contain three prominent MAPK systems: the extracellular signal–regulated kinase (ERK) 1/2, c-Jun N-terminal kinase (JNK) 1/2/3, and p38 MAPK *α*, *β*, *δ*, and *γ* pathways. In addition to their activation motifs, structural and functional similarity is utilized to categorize ERK, JNK, and p38 isoforms [45, 46]. The activation of ERK1/2 occurs when growth factors, hormones, and proinflammatory stimuli are present. Additionally, JNK1/2/3 and p38 MAPK*α*, *β*, *δ*, and *γ* are activated in response to proinflammatory stimuli, as well as cellular and environmental stressors [43].

#### 1.2.1. MAPK Mechanism in Inflammation

In inflammation, the MAPK pathway begins when the extracellular stimuli such as stress, growth factors, pathogens, and cytokines (TNF-*α* and IL-1) bind with unambiguous receptors present on the surface of the cell. These receptors are G-protein coupled receptors (GPCRs), TLRs, and tyrosine kinases (RTKs). MAPK signaling cascade is initiated by the recruitment of the adaptor proteins such as SOS, Geb2, and TRAF, and this recruitment happens upon the receptor activation [47, 48]. Then adaptor proteins promote the conversion of GDP to GTP which leads to the activation of GTPase Ras. This activated Ras further interacts with Raf which is serine or threonine protein kinase. This cascade lingered as the Ras phosphorylates and MEK (MAP2K) activates which further phosphorylates as well as activates the ERK (MAPK). These activated ERKs transfer to the nucleus and phosphorylate different transcription factors such as c-Jun, c-Fos, and Elk-1 which leads to proinflammatory gene expression [49]. In another subtype of MAPK, such as JNK pathway different MAP3Ks (MLKs and ASK1) activate and phosphorylate MKK4/7 (MAP2Ks) which further activate JNK. This activated JNK transfers to the nucleus and phosphorylates the transcription factors (Elk-1, ATF2, and C-Jun) which results in the gene expression which is involved in apoptosis and inflammation [50]. Likewise, in the p38 pathway, different proteins (MAPK3s) activate MKK3/6 or MAP2K, and it phosphorylates as well as activates p38 MAPK. This activated p38 moves to the nucleus and different transcription factors like MEF2, CREB, and ATF2 phosphorylate by it and genes involved in the inflammation expressed [47, 50]. Expression of enzymes, cytokines, and chemokines increased because all the activated MAPKs enhance the ability of transcription factors to bind with DNA by their phosphorylation. This increases the production of proinflammatory cytokines that amplify the inflammation [51].

#### 1.2.2. ERK and P38 MAPK Pathways

Within the plasma membrane (PM), a ligand binds to an RTK, which then activates the small G-protein Ras and commences the classical activation of ERK1 and ERK2 isoforms. This process is known as the Ras-activation pathway. An MAP3K called Raf, which is responsible for phosphorylating threonine and tyrosine residues in the TEY motif, is activated by Ras for the process to continue. Upon receiving this reaction, Raf causes MAP2K MEK to become activated, which in turn phosphorylates MAPK ERK1/2 [52]. The Ras/Raf/MEK/ERK1/2 pathway is prone to inhibition by dual-specificity MKPs. Although MKP3 and MKP-X specifically target ERK, MKP2/4 also dephosphorylates ERK1/2 and additional MAPKs [38]. An additional enzyme responsible for stimulating the Ras G-protein is tyrosine phosphatase SHP2 [45, 46]. p38 MAPK isoforms are stimulated in inflammatory responses by cytokines and stress. These isoforms are encoded in numerous genes and display a wide range of tissue expression patterns [44, 53, 54]. The process of successive activation of p38 MAPKs is similar to that of ERK isoforms. A typical activation occurs when Rho proteins or TRAF (tumor necrosis factor receptor-related factor) 2/3/6 stimulate an MAP3K like MEKK1, ASK1, or TAK1 in response to cytokines or stress. Following MAP2K phosphorylation, MAP3K, specifically MKK3 or MKK6, phosphorylates the TGY motif on p38 isoforms [54, 55]. Several protein phosphatases with dual specificity, including MPK2/4, dephosphorylate the *α*, *β*, *δ*, and *γ* subunits of the p38 MAPKs; this process also inhibits ERK. Phosphatases MPK5/7 are capable of dephosphorylating both JNK and p38; however, MPK1 exhibits a more pronounced affinity for p38 [56].

## 2. Licorice Derivatives and NF-*κ*B Pathway

Many studies highlighted that phytocomplexes from licorice play an important role in the many biological activities such as antioxidant and anti-inflammatory in the different animal models as well as cell lines. Even though *G. glabra* roots were the most utilized part, the aerial parts also have many bioactive compounds, for instance, isoquercitrin, prunetin, formononetin, dihydrostilbenes, licoflavanone, genistein, lupiwighteone, wighteone, and 6-prenylnaringenin [57]. These licorice derivatives prevent the I*κ*B*α* (an inhibitory protein) degradation which inhibits the activation of NF-*κ*B. As shown in Table 1, a study was conducted to investigate the anti-inflammatory activity of the purified compounds and extract in the RAW 264.7 murine macrophages. Isoflavones, a bioactive compound present in the licorice, exhibited an anti-inflammatory activity without affecting the viability of cells. It also notably reduces the proinflammatory COX-2/iNOS expression levels and cytokines. There was also modulation in the NF-*κ*B/MAPK cell signaling pathway. Licoflavanone decreases the activation of the NF-*κ*B transcription factor. This compound reduces the translocation of this transcription factor into the nucleus and notably reduces the target genes, COX-2 and iNOS [58]. Glycyrrhizin is a bioactive component present in the licorice and also suppresses expression of microglial marker IBA-1 and the production of NO. The expression of inflammatory cytokines such as IL-6, TNF-*α*, and IL-1*β* was remarkably reversed, and glycyrrhizin also reduced the activation of the TLR4-NF-*κ*B pathway. Expression of p65-NF-*κ*B protein and TLR4 was also nullified in the nucleus after the treatment of glycyrrhizin [4]. Licorice contains flavone compound called Liquiritin which showed the potential as antioxidant, anti-inflammatory, antitussive effect, and also has therapeutic potential against psoriasis. In the rat model with psoriasis after the treatment of liquiritin NF-*κ*B was reduced. The results showed that liquiritin modulates the NF-*κ*B signaling pathways by inhibiting the phosphorylation of NF-*κ*B p65 [59]. GA and its amalgamation with bacteria decreased the contrary effect in hepatocarcinogenesis rats by various methylomic changes in NF-*κ*B and STAT-3 genes by 76% and 83%, respectively. The methylation activity in coding sequence of NF-*κ*B increases significantly in the group of rats treated with GA [60].

Another study observed that Glycyrrhiza (GL) significantly decreases the proinflammatory factor production such as IL-1*β*, IL-6, and TNF-*α*. NF-*κ*B and MAPK activation and expression of TLR4 were drastically decreased. Moreover, LicoA also inhibits inflammation and lung damage by inhibiting the expression of TLR4 as well as the activation of MAPK and NF-*κ*B cell signaling pathways. GL significantly reduces the phosphorylation of p65 protein which is very important to make heterodimers, translocation, and activation in the nucleus [61]. A study on temporomandibular joint osteoarthritis (TMJOA) revealed that glycyrrhizin as the novel therapeutic drug has the potential to decrease the degradation and pathological changes of TMJ cartilage by modifying HMGB1-RAGE/TLR4-NF-*κ*B/AKT signaling pathway. Glycyrrhizin modified the pathway inhibiting the phosphorylation of HMGB1, so the HMGB1 (proinflammatory) expression in the TMJOA decreased [62].

Psoriasis is characterized by keratinocyte hyperproliferation which is an immune-mediated inflammatory disease. It can be triggered by stimuli from the environment, genetic predisposition, and immune system dysfunction. Glycyrrhizin inhibited the phosphorylation and dysregulation of the NF-*κ*B pathway [70]. Extract of licorice roots contains the isoflavone called glabridin (Gla) which has different therapeutical effects such as antiatherosclerotic, antiobesity, and antioxidant. Alcoholic liver disease (ALD) is a liver disease which occurs due to the long-term consumption of alcohol. A research was designed to explore the Gla effect in the C57BL/6J mice and HepG2 cells treated with ethanol. Gla lessened the liver injury induced by the ethanol including reducing the lipid accumulation and vacuolation. Levels of the inflammatory cytokines in the serum and nuclear translocation of NF-*κ*B were decreased and enhances the nuclear translocation of nuclear factor (erythroid-derived 2)-like 2 (Nrf2). Gla plays a positive role in alleviation of ALD damage through the MAPK/Nrf2/NF-*κ*B pathway and can also be used as a novel product and drug to notably alleviate ALD [60].

Wound healing is a complex and systemic cellular and molecular process. A byproduct from glycyrrhizic acid known as dipotassium glycyrrhizinate (DPG) has different biological effects (antiallergic, antibacterial, antioxidant, antiviral, antitumoral, and anti-inflammatory). A study explored that the DPG decreased inflammation by reducing the proinflammatory cytokines (Cox-2, TNF-*α*, Irak-2, Il-8, Il-1, and NF-*κ*B) expression. It was concluded that DPG attenuates the process of inflammation by supporting wound healing by modulating the mechanism and cell signaling pathway specifically the anti-inflammatory pathway. It modulates the expression of the anti-inflammatory cytokine, tissue re-epithelialization, and angiogenesis all of which contribute to tissue remodeling [65].

Extensively distributed nuclear transcription factor NF-*κ*B has many functions. The transcription of a gene can be started by combining it with a certain sequence found in the promoter or enhancer regions of that gene [66]. In the cytoplasm, NF-*κ*B and I*κ*B typically form an inactive complex [71]. I*κ*B is phosphorylated which is processed by ubiquitination when it is exposed to signals of extracellular stimulation. The target gene's *κ*B will bind with released NF-*κ*B P50/P65 in the nucleus, causing a conformational change in the DNA and subsequently starting or accelerating the target gene's transcription. This process is dependent on the action of 26S protease [63, 69]. An investigation revealed that in MI rats, isoquercetin markedly reduced the expression of TLR4 and NF-*κ*B proteins [65, 71]. In another study, effect of formononetin (a bioactive component present in the aerial part of the licorice) on NF-*κ*B signaling pathway*–*related proteins involved in gastric ulcers was detected. Notable downregulation of ratio of p-P65/P65 (protein in NF-*κ*B) and upregulation of ratio of p-I*κ*Ba/I*κ*Ba were observed in the rat groups treated with formononetin [69].

## 3. Licorice Derivatives and MAPK Cell Signaling Pathway

The MAPK cell signaling pathway is activated by infection. The MAPK cascade pathway has an important role in transferring signals to the intracellular environment and finely regulating several activities of cells. Based on the respective terminal MAPK constituents, three distinct MAPKs have been recognized, for instance, ERK and JNK. Licorice derivatives inhibited the phosphorylation of MAPKs specially p38 and JNK that are often activated in the result of inflammation. Procytokines and other inflammatory mediator production is reduced as the result of the inhibition of phosphorylation [63, 72, 73]. The MAPK cascade plays a vigorous role in infections such as influenza and foot-and-mouth disease [74, 75]. The MAPK p38 cascade pathway is a crucial regulator of inflammatory responses [76–79]. As in Table 2, a study demonstrated that glycyrrhizin decreased p38 phosphorylation. Collectively, data-directed glycyrrhizin inhibited porcine epidemic diarrhea virus (PEDV) infection and reduced the secretion of proinflammatory cytokine through the HMG B1/TLR4-MAPK p38 pathway [80].

A previous study showed that isoliquiritigenin (ISL) inhibited the IL-1*β* secretion and the pore-forming protein activation (gasdermin D, GSDMD). Inflammatory factors (IL-6, iNOS, TNF-*α*, and COX-2) were inhibited by ISL. ISL has anti-inflammatory effects because it inhibits the MAPK signaling and NF-*κ*B pathways. In a study, it was concluded that ISL can be used as a potential drug for the treatment of tuberculosis [72]. ERK1/2, JNK 1/2, and p38 MAPK are the major components of the MAPK pathway in platelets. An investigation revealed that glabridin (at 25 and 40 μM) suppressed the phosphorylation of the three previously described MAPKs in response to collagen, suggesting that MAPK signaling plays a role in glabridin's suppression of platelet activation. MAPK and PI3K/Akt/GSK3*β* pathway activation is prevented and effectively inhibited by the glabridin which leads toward the reduction in Ca2^+^ mobilization and to inhibit the aggregation of platelets. Glabridin acts as a therapeutic agent in thromboembolic disorders [89]. Licochalcone is a bioactive compound present in licorice that reduced the level of TNF-*α* and cytokines IL-6 and also inhibited the phosphorylation in MAPKs p38 MAPK and Erk 1/2 [80].

In the ERK/MAPK signaling pathway, mitogen extracellular kinase 1/2 (MEK1/2) and ERK1/2 are the core proteins. The activation of these proteins is related to the cell cycle, proliferation, and apoptosis. ERK/MAPK signaling pathway activation is determined by the increased level of phosphor-MEK1/2 (p-MEK1/2) indicated and phosphor-ERK1/2 (p-ERK1/2). The downstream of the ERK/MAPK signaling pathway is located by the prostaglandin-endoperoxide synthase 2 (PTGS2), and the expression reflects that either pathway is activated or inhibited. The levels of PTGS2, p-ERK1, and p-MEK1 were significantly reduced after the treatment with quercetin [83]. Different proteins such as ERK, p38, and JNK are parts of the MAPK family. All these proteins are activated by different extracellular stimuli and perform key roles in melanin synthesis. For instance, microphthalmia-associated transcription factor (MITF) expression is inhibited by the phosphorylation of ERK and thereby synthesis of melanin decreased. Results of this study showed that the melanogenesis in B16 cells is suppressed by the glabridin, and the p38 MAPK signaling pathway is involved in it because the upregulation of p-38 is reversed by the glabridin [85].

Based on the ability of Gancaonin to reduce the expression of proinflammatory cytokines and COX-2, immunoblotting was used to assess the MAPK/NF-*κ*B signaling pathway's protein expression level, which is linked to inflammation, to better understand the mechanism underlying the anti-inflammatory actions. Consequently, ERK and p38 phosphorylation was upregulated in A549 cells stimulated by LPS alone; however, phosphorylation of ERK and p38 was successfully inhibited in the group that received pretreatment with 10–40 μM Gancaonin N. It was verified that Gancaonin N reduced the nuclear translocation of NF-*κ*B p65 in A549 cells in the nuclear fraction. Gancaonin N inhibits the MAPK, hence controlling the expression of proinflammatory cytokines and inflammatory mediators like COX-2 [90]. The mRNA levels of p65, JNK, and p38 were reduced in human osteosarcoma cells on the treatment of prunetin [88].

## 4. Limitation and Toxicity of Licorice

According to the United States Food and Drug Administration (21 CFR 184.1408), licorice and its derivatives are generally recognized as safe (GRAS) for use in food. FAO/WHO Expert Committee on Food Additives (JECFA) concluded that the 100 mg/day consumption of glycyrrhizin is safe for human health. The list of GRAS compounds published by the Flavour and Extract Manufacturers Association (FEMA) also includes licorice root, extract, powder, and ammoniated glycyrrhizin in it. Licorice is regarded by the Food Additive and Contaminants Committee of the United Kingdom and the Council of Europe as a naturally occurring plant product that should be used in small amounts as a food additive. The goal is to keep consumption of licorice within the naturally occurring range of glycyrrhizin levels in foods [91]. Glycyrrhizin was limited by these groups to fewer than 50 parts per million. The FAO/WHO Expert Committee on Food Additives convened a combined meeting in 1977, but the judgment on an acceptable daily intake is pending. The committee evaluated the safety of glycyrrhizin and concluded that a daily dose of 100 mg (about 2 mg/kg) is unlikely to induce adverse effects in most persons. The committee acknowledged that persons with high susceptibility may have physiological effects at intakes lower than the recommended dose (100 mg/day). The committee stated that consumers who consume licorice sweets or herbal tea may be exposed to glycyrrhizin at levels above 100 mg/day [92]. People who consume amounts>100 mg/day from different sources such as herbal tea of licorice or confectionery may experience some physiological changes [51]. The extract of the licorice has glycyrrhizin approximately 10–20 percent, and the dose of 2 to 4 mL provides 200–800 mg glycyrrhizin [93]. A standard dose of licorice flavonoid oil is 300 mg thrice a day. Ingestion of licorice root (>3 g/day) for consecutive 6 weeks can be hazardous to human health as GA has aldosterone-like effects. It can cause hypertension, water and sodium retention, and hypokalemia [81]. Moreover, licorice toxicity depends on different factors such as sex, age, metabolism, comorbidities, and concomitant medication use [94]. However, excessive daily intake of easily accessible items might increase the risk of exceeding the safety threshold and developing hazardous signs or symptoms (flavored sweets or beverages). De-glycyrrhizinated licorice was created lately to lessen the possibility of licorice poisoning as a negative consequence [94–96].

## 5. Conclusion

The emerging field of clinical medicine is focusing on the therapeutic potential of natural chemicals and medicinal plants, with licorice being a prominent herb used in traditional Chinese medicine. Licorice root contains various bioactive compounds such as glycyrrhizin and flavonoids, which have antiviral, anticarcinogenic, anti-inflammatory, and immunological properties. These components make licorice beneficial for treating respiratory, digestive, and immunological conditions without significant negative effects reported. Inflammation is a common pathophysiological mechanism in chronic diseases like cancer, inflammatory bowel disease, heart disease, and diabetes. The inflammatory response involves the generation of inflammatory mediators, recruitment of inflammatory cells, and stimulation of inflammatory pathways. The NF-*κ*B and MAPK pathways play crucial roles in regulating inflammation, with licorice compounds demonstrating anti-inflammatory effects by modulating these pathways. Studies have shown that licorice components like glycyrrhizin, liquiritin, and LicoA can inhibit proinflammatory factors, reduce NF-*κ*B and MAPK activation, and modulate signaling pathways involved in inflammation.

Licorice contains the bioactive components such as glycyrrhizin, liquiritin, glabridin, and many others which play a significant role in inhibiting the NF-kB cell signaling pathway. These compounds are good in inhibition of phosphorylation as well as degradation of I*κ*B*α* that leads to the reduction in the translocation of proteins in the nucleus which in result prevent the NF-*κ*B activation. This NF-kB suppression results in reduction of proinflammatory (mediators such as COX-2, TNF-*α,* and IL-6) expression. Therapeutic agents in the licorice have also been found to attenuate the MAPK cell signaling pathway. The major key proteins such as JNK, ERK, and p38 inhibited by the licorice compounds, and these proteins are involved in the inflammatory response, By inhibiting MAPK pathway, licorice reduced the inflammation.

Licorice shows promise as a natural remedy for various inflammatory conditions by targeting key pathways involved in the inflammatory response. For the future suggestion, there is a need to explain in detail exact molecular mechanisms underlying the anti-inflammatory effects of licorice at the cellular level; also there should be efficacy trials to check the bioavailability and pharmacokinetics of the licorice compounds. All of the studies reviewed in this article are rat trials or cell line and there should be clinical trials to assess their efficacy in humans.

## Figures and Tables

**Table 1:** Effect of licorice on NF-*κ*B pathway.

Sr. #	Disease	Type of licorice	Compound	Cell line/tissue	Effect	Reference
1.	Inflammation adaptive response generated by harmful stimuli or injury	*Glycyrrhiza glabra*	Phenolic compounds and flavanones	Murine macrophages RAW 264.7 cell line	Inhibition of the cascade of the main MAPKs: ERK1/2, JNK, and p38	[58]
2.	Chronic inflammatory pain caused by noxious stimulus		Glycyrrhizin	Murine BV2 microglial cells	Elevation of HMGB1, TLR4, and p65 NF-*κ*B expression	[4]
3.	Psoriasis, immune-mediated genetic inflammatory disease		Liquiritin	HaCaT cells	Strongly inhibit the NF-*κ*B p65 phosphorylation. TNF-*α* stimulated c-Fos phosphorylation was also reduced	[59]
4.	Hepatocarcinogenesis, most aggressive liver cancer		18*β*-Glycyrrhetinic acid	Liver tissue	Significantly lowers the gene expression of STAT-3 and NF-*κ*B Methylomic changes in the coding sequence of NF-*κ*B	[60]
5.	Acute lung injury, a type of lung disease caused by inflammation	*Glycyrrhiza glabra*	Glycyrrhizic acid, liquiritin, liquiritigenin, isoliquiritigenin, monoammonium glycyrrhizinate, LicoA, and glycyrrhetinic acid	THP-1 cells	Suppression of NF-*κ*B activation	[61]
	Temporomandibular joint osteoarthritis, a degenerative joint disease caused by cartilage degradation		Glycyrrhizin	Chondrocytes and disc cells	Downregulation of IL-6 and MMP-3 Reduction in the activation of both AKT and NF-*κ*B p65	[62]
8.	*Mycobacterium tuberculosis*–induced inflammation or tuberculosis (TB)	*Glycyrrhiza uralensis Fisch, Glycyrrhiza inflata,* or *Glycyrrhiza glabra* L.	Isoliquiritigenin	Raw264.7 or primary peritoneal macrophage cells	Inhibit the phosphorylation of p65 subunit of NF-*κ*B	[63]
9.	Nonalcoholic steatohepatitis progression of nonalcoholic fatty liver disease (NAFLD)	*Glycyrrhiza uralensis Fisch*	Glycyrrhiza	Bone marrow–derived macrophages (BMDMs)	Reduced colon NF-*κ*B gene expression	[64]
11.	Alcoholic liver disease (ALD), inflammation caused by excessive alcoholic consumption		Glabridin	HepG2 cells	Inhibit p-NF-*κ*B nuclear translocation	[65]
12.	Wound healing	*Glycyrrhiza glabra*	DPG	Cutaneous tissue	TNF_, IL-1, IL-6, and NF-*κ*B levels were significantly reduced	[66]
13.	Allergy rhinitis (AR), an airway inflammatory disease		Glycyrrhizin	HepG2 cells	NF-*κ*B was blocked and the nasal allergic inflammation was reduced	[67]
14.	Acute myocardial infarction (AMI), an inflammatory condition		Isoquercetin	Sprague-Dawley (SD) rats	Expression of TLR4 and NF-*κ*B protein was significantly suppressed	[68]
15.	Gastric ulcer, a gastrointestinal disease		Formononetin	Sprague-Dawley (SD) rats	Significant down regulation of p-P65/P65 and upregulation of the ratio of p-IkBa/IkBa	[69]

Abbreviation: TNF, tumor necrosis factor.

**Table 2:** Effect of licorice on MAPK pathway.

Sr. #	Disease	Type of licorice	Compound	Cell line/tissue	Effect	Reference
1.	*Mycobacterium tuberculosis*–induced inflammation or tuberculosis (TB)		Isoliquiritigenin	Murine macrophage cell line Raw264.7	Prevents/stops activation of ERK and JNK MAPK signaling pathway (earlier phase of TB infection)	[63]
2.	Porcine epidemic diarrhea (PED) caused by virus (PEDV)		Glycyrrhizin (GLY)	Vero cells	Inhibit p38 phosphorylation and reduce proinflammatory cytokine mRNA level	[79]
3.	Alcoholic liver injury, inflammation caused by excessive alcoholic consumption	*G. uralensis*	GA		Inhibiting the expression of key targets of the MAPK signaling pathway	[61]
4.	Pathological platelet activation		Glabridin		Lowers activation of MAPK	[80]
5.	Alcoholic liver disease (ALD) induced by the chronic alcoholic consumption		Glabridin	HepG2 cells	Suppress the p38 MAPk signaling pathway	[81]
6.	Epilepsy, complex neurological syndrome induced by abnormal neuron discharge		Glycyrrhizin	Brain tissues	Levels of p38MAPK and p-p38MAPK were significantly downregulated	[82]
7.	Neuroinflammation progression to neurological and neuropsychiatric disorders		Licochalcone	Microglia cells	Decreased phosphorylation of p38 MAPK	[83]
8.	Gastric cancer		Quercetin	Human gastric cancer cell line MKN-45	Reduce levels of ERK1, p-MEK1, PTGS2, and p-ERK1	[84]
9.	Melanogenesis		Glabridin	B16 cells	Downregulation of p38 MAPK signaling	[85]
10.	Acute pneumonia, an inflammatory disease caused by several pathogens	*Glycyrrhiza uralensis*	Gancaonin N	eRAW264.7 cell line	Inhibited the phosphorylation of ERK and p38 MAPK	[86]
11.	Oral squamous cell carcinoma (OSCC) generally known as oral cancer caused by tobacco and alcohol	*Glycyrrhiza glabra* and *Glycyrrhiza uralensis*	Semilicoisoflavone B (SFB)	Human OSCC cell lines SAS, HSC3M3, OECM-1, and SCC9	Reduction of MAPK along with Ras/Raf/MEK signaling	[87]
12.	Antichronic nonbacterial prostatitis	*Glycyrrhiza uralensis*	Glycyrrhisoflavone	Prostate tissues	Reduction of interferon-IP-10, IL-8, and other inflammatory factors in the MAPK	[84]
13.	Psoriasis, immune-mediated genetic inflammatory disease		Glycyrrhizin	Human keratinocyte (HaCaT) cell line	Downregulation and inhibition of NF-*κ*B	[61]
14.	Osteosarcoma type of cell cancer develops in the osteoblast cells		Prunetin	MG-63 cells	Reduction in expression of p56 and p38 proteins	[88]

## Data Availability

Data will be available on demand from the corresponding author.
